# Metabolic manipulation in chronic heart failure: study protocol for a randomised controlled trial

**DOI:** 10.1186/1745-6215-12-140

**Published:** 2011-06-06

**Authors:** Roger M Beadle, Lynne K Williams, Khaild Abozguia, Kiran Patel, Francisco Leyva Leon, Zaheer Yousef, Anton Wagenmakers, Michael P Frenneaux

**Affiliations:** 1 University of Aberdeen, School of Medicine and Dentistry, Polwarth Building, Foresterhill, Aberdeen, AB25 2ZD, UK; 2 Division of Cardiology, Toronto General Hospital, University Health Network, Toronto, M5G 2C4, Ontario, Canada; 3 University of Birmingham, Edgbaston, Birmingham, B15 2TT, UK; 4 University Hospital of Wales, Heath Park, Cardiff, CF14 4XW, UK

## Abstract

**Background:**

Heart failure is a major cause of morbidity and mortality in society. Current medical therapy centres on neurohormonal modulation with angiotensin converting enzyme inhibitors and β-blockers. There is growing evidence for the use of metabolic manipulating agents as adjunctive therapy in patients with heart failure. We aim to determine the effect of perhexiline on cardiac energetics and alterations in substrate utilisation in patients with non-ischaemic dilated cardiomyopathy.

**Methods:**

A multi-centre, prospective, randomised double-blind, placebo-controlled trial of 50 subjects with non-ischaemic dilated cardiomyopathy recruited from University Hospital Birmingham NHS Foundation Trust and Cardiff and Vale NHS Trust. Baseline investigations include magnetic resonance spectroscopy to assess cardiac energetic status, echocardiography to assess left ventricular function and assessment of symptomatic status. Subjects are then randomised to receive 200 mg perhexiline maleate or placebo daily for 4 weeks with serum drug level monitoring. All baseline investigations will be repeated at the end of the treatment period. A subgroup of patients will undergo invasive investigations with right and left heart catheterisation to calculate respiratory quotient, and mechanical efficiency. The primary endpoint is an improvement in the phosphocreatine to adenosine triphosphate ratio at 4 weeks. Secondary end points are: i) respiratory quotient; ii) mechanical efficiency; iii) change in left ventricular (LV) function.

**Trial Registration:**

ClinicalTrials.gov: NCT00841139

ISRCTN: ISRCTN2887836

## Background

Despite recent advances in the treatment of heart failure (HF), it remains a condition with significant morbidity and mortality having a 5 year mortality rate that rivals most cancers[1]. In the developed world, heart failure affects 1-2% of the general population [2], causing about 5% of all adult hospital admissions, and complicating a further 10-15%[3]. Current pharmacological therapy centres on the management of symptoms with diuretics and neurohormonal modulation with angiotensin converting enzyme inhibitors and β-blockers. Energy deficiency plays an important role in the pathophysiology of HF and is held to be a promising target for HF therapy. Numerous studies support the finding of decreased cardiac energy levels and flux as a consistent feature of HF[4,5].

The main determinants of cardiac energetics and energy availability are substrate utilisation. The normal heart relies mainly on fatty acids for its energy requirements, accounting for up to 70% of the total energy requirement in the fasting state[6]. Glucose and other carbohydrates such as lactate make up much of the remainder. Mitochondrial phosphorylation represents the major route of energy generation. The heart is a metabolic omnivore and can adapt to utilise various substrates depending on requirements, but each substrate varies in its energy cost. Fatty acid oxidation (FAO) requires greater oxygen for a given quantity of adenosine triphosphate (ATP) synthesis than the use carbohydrates. In HF, there is typically a down regulation of fatty acid metabolism with preserved or increased glucose uptake, but often a relative block of entry of pyruvate in to the tricarboxylic acid cycle (TCA cycle).

These metabolic changes contribute to the energy starvation state found in heart failure of all aetiologies. Phosphorus-31 (P-31) magnetic resonance spectroscopy (MRS) is used to non-invasively estimate ATP and phosphocreatine concentration and the PCr/ATP ratio. Phosphocreatine is an important short-tem energy store that maintains a high phosphorylation potential under conditions of rapid increased energy demand such as exercise. ATP levels are maintained at steady concentration to maintain energy for muscle contraction and normal cellular function. The myocardial PCr/ATP ratios is reduced in HF and correlates with New York Heart Association (NYHA) class[5].

Perhexline maleate is a metabolic modulating agent that gained popularity in the 1970's as an antianginal therapy until it was linked to both peripheral neuropathy [7] and hepatotoxicity[8]. It has since been shown that the risk of toxicity can be dramatically reduced by maintaining plasma concentrations in an established normal range of between 0.15 and 0.6 mg/L[9]. In the isolated rat heart, perhexiline acts, at least in part, by shifting myocardial substrate utilisation from fatty acids to carbohydrates through the inhibition of carnitine palmitoyltransferase-1 (CPT-1) and, to a lesser extent, carnitine palmitoyltransferase-2 (CPT-2). These mitochondrial enzymes act to facilitate the entry of medium and long chain fatty acids into the myocytes as their main energy source. Their inhibition causes a reciprocal increase in glucose and lactate utilisation and thus an improvement in myocardial efficiency and energy status by forcing the omnivorous myocytes to metabolise carbohydrate and reduce the net oxygen consumption.

A growing body of studies support the use of P-31 MRS to measure this improvement in myocardial energetics as a response to treatment. Fragasso et al [10] have demonstrated an increase in cardiac PCr/ATP ratio with the metabolic modulating agent trimetazidine in subjects with heart failure. Our group has recently shown an improvement in cardiac energetics using perhexiline in patients with hypertrophic cardiomyopathy[11].

## Methods

### Hypothesis

Treatment with perhexiline increases cardiac PCr/ATP ratio, increases cardiac respiratory quotient (indicating relatively greater carbohydrate utilisation), and improves LV mechanical efficiency and left ventricular function in patients with dilated cardiomyopathy.

### Study Design

This is a multicentre prospective, randomised, double-blind, placebo-controlled trial of 50 subjects with dilated cardiomyopathy (defined as an ejection fraction (EF) <40% in the absence of evidence of coronary artery disease) who are stable on conventional heart failure therapy. Inclusion and exclusion criteria are detailed in Table 1.

**Table 1:** Inclusion and exclusion criteria.

*Inclusion Criteria*
Non-ischaemic, dilated cardiomyopathy
Symptomatic despite optimally-tolerated medical therapy
Impaired left ventricular systolic function (LVEF < 40%) as assessed by resting echocardiography
*Exclusion Criteria*
History of liver disease or liver function test measurements greater than twice the upper normal limit
Concomitant use of amiodarone, quinidine, haloperidol or selective serotonin re-uptake inhibitors that inhibit the CYP2D6 enzyme
Pre-existing evidence of peripheral neuropathy
Women of child bearing potential
Patients with any contraindication to magnetic resonance imaging such as an implantable cardiac device or claustrophobia
Obesity (BMI > 35)
Obstructive sleep apnoea

### Baseline Studies

**Figure 1:**
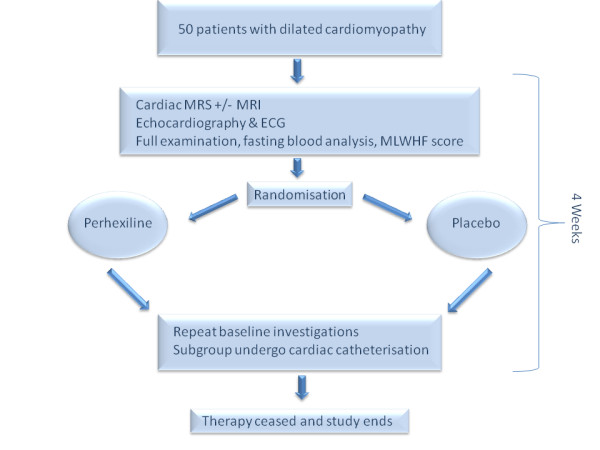
**Summary of study timeline**. Following baseline investigations, 50 patients will be randomised to recieve 200 mg of perhexiline or matched placebo for one month. At one month all baseline tests will be repeated and a subgroup will undergo cardiac catheterisation.

All patients will undergo a baseline visit (see flowchart - Figure 1) during which the following will be performed; i) detailed review of past medical history, drug history and tobacco and alcohol consumption; ii) clinical examination of all systems; iii) assessment of NYHA class from history; iv) completion of a Minnesota Living with Heart Failure (MLWHF) questionnaire; v) measurement of height and weight; vi) 12-lead electrocardiogram (ECG); vii) office brachial blood pressure and heart rate measurement following 15 minutes rest; viii) collection of fasting serum and plasma for haematological and biochemical analysis including glucose and liver function tests; ix) storage of serum and plasma at -80°c for future assay of biomarkers of heart failure such as pro-terminal brain natriuretic peptide (BNP); x) transthoracic echocardiography to determine left ventricular size and function; xi) cardiac magnetic resonance spectroscopy to determine the cardiac PCr/ATP ratio;

Baseline tests will be repeated in full after completing one month of treatment with perhexiline or placebo. A willing subgroup of patients will undergo right and left heart catheterisation for invasive haemodynamic measurements and cross heart sampling. Those subjects taking part in the invasive subgroup will also undergo cardiac magnetic resonance imaging (CMR) for accurate estimation of left ventricular cardiac mass.

Female subjects of child-bearing potential will be excluded from the study.

### Cardiac Magnetic Resonance Spectroscopy

Cardiac high-energy phosphate metabolism will be measured using P-31 MRS on a 3-Tesla Philips (Philips Healthcare, Reigate, Surrey) Achieva whole-body magnet using a linearly polarized transmit and receive P-31 coil with a diameter of 14 cm. Localization will be achieved by image selected in-vivo spectroscopy (ISIS) volume selection. The participants will be positioned supine with the coil directly over the precordium. The coil will be secured in place by straps around the upper body and coil. The participants will then be positioned inside the magnet with the center of the coil at the isocenter of the magnet. Survey images will be obtained to check the position of the coil. The subjects and/or the coil will be repositioned if required to ensure that the distance between coil and septum and apex of the heart is minimized and maximize signal strength. Localized iterative 1^st ^order shimming will be performed including the entire heart using the unsuppressed water signal acquired with the body coil as reference. The shimming process involves an automated Hydrogen-1 spectral acquisition to test the quality of the shim that is expressed as a full width at half maximum (FWHM). This is repeated until a FWHM of less than 40 Hz is achieved. A short axis cine scan will be acquired to calculate the trigger delay for ECG triggering and to check quality of shimming and F_0 _determination. The trigger delay will be calculated such that the spectra is acquired in the diastolic period when the heart is a still as possible. The 3-dimensional ISIS voxel of acquisition will be planned to include most of the septum and apex of the heart within the shimmed area. Care will be taken to minimize blood contamination from the right ventricle, liver and skeletal muscle as much as possible. The voxel size will be kept constant at 89.54 ml (44 × 55 × 37 mm^3^) to allow comparisons between different subjects and scans. Following this the P-31 spectrum will be acquired with a repetition time of 10000 ms and 512 averages. A repetition time of 10000 ms has found to be optimal for adequate reduction of saturation effects without increasing the scan time greatly. The total scan time will be 23 minutes. The spectra will be analyzed and quantified on the java based Magnetic Resonance User Interface (jMRUI) software. Post-processing will be performed with 15 Hz Gaussian line broadening and Fourier transformation. Phase correction will be performed with PCr peak as the reference peak. Quantification will be performed with AMARES (Advanced Method for Accurate, Robust and Efficient Spectral fitting), time domain fitting program, which involves selecting peaks and defining their line width[12]. The concentrations of PCr, ATP (γ, α and β) and 2,3-diphosphoglycerate (2,3-DPG) will then be calculated as the area under the peaks. PCr/ATP ratio will be determined after correcting the γ ATP peak for blood contamination as described previously based on the quantity of 2,3-DPG in the derived spectrum[13]. A single, blinded operator experienced in the technique will perform analysis.

### Cardiac Magnetic Resonance Imaging

Cardiac magnetic resonance imaging will be performed on the same 3 Tesla Philips Achieva system using a cardiac coil with patients in the supine position. Serial contiguous short axis cines will be piloted from the vertical long axis and horizontal long axis images using standard manufacturer supplied sequences. Analysis will be performed offline (Philips ViewForum Ver 4.1) by a single blinded observer for the assessment of ventricular volumes, ejection fraction and left ventricular mass.

### Echocardiography

A standard transthoracic echocardiogram (Vivid 7, GE Vingmed Ultrasound, Horten, Norway) will be performed with the subject in the left lateral decubitus position by a single experienced echocardiographer using second harmonic imaging and an M3S multi-frequency transducer. All parameters will be measured in triplicate and averaged as per the recommendations of the American Society of Echocardiography[14]. Analysis will be performed offline by a single blinded observer using an EchoPAC workstation (GE Vingmed Ultrasound, Horten, Norway). Ventricular dimensions, wall thickness, chamber volume, stroke volume and ejection fraction will be determined by standard methods[15]. Resting left ventricular diastolic function will be determined using standard techniques[16]. Peak systolic (S'), early diastolic (E') and late diastolic (A') mitral annular velocities will be measured at end expiration at the septal, lateral, inferior and anterior left ventricular walls with real time pulsed wave tissue Doppler[17].

Greyscale images for 2-dimensional left ventricular strain will be acquired in cine-loop format in triplicate from the apical 4-, 2- and 3-chamber views and parasternal short axis views at basal, papillary and apical ventricular levels at end expiration at frame rates >70 Hz for offline analysis using commercially available software (Speqle Tracking, GE Healthcare, United Kingdom). The endocardial border will be manually tracked at end-systole and the software generates a region of interest over the myocardium. This enables frame-to-frame tracking of ultrasonic speckles that change position according to surrounding tissue motion throughout the length of the cardiac cycle. Peak systolic velocities, strain, strain rate, rotation and twist will be measured for each myocardial segment in triplicate and averaged.

### Invasive testing

Patients will undergo cardiac catheterisation via the femoral artery and either the femoral or internal jugular vein following appropriate consent. A 7F sheath will be placed in the vein and artery. A Cordis-Webster (Cordis Webster, Inc., Baldwin Park, CA, USA) 7F coronary sinus (CS) thermodilution catheter will be guided into the coronary sinus and 50/50 saline/contrast injections will allow visualisation and confirmation of its position within the right atrium and CS. All catheters will be calibrated prior to utilisation. The catheter is connected to both a Medtronic (Medtronic Inc., 7000 Central Avenue NE, MN, USA) temporary pacemaker 5348 and to a specialised Wheatstone bridge coupled to a bank of isolated direct coupled bioamplifiers (Coulbourn Instruments, Whitehall, PA). The output is subsequently digitised using a Dataq DI720 data acquisition system and both displayed and stored on a laptop computer using Windaq software (Dataq, Akron, OH). The atria will then be paced via electrodes on the catheter in the coronary sinus.

A 7F dual-field conductance catheter (CA-71103-PL catheter, CD Leycom, The Netherlands) will then guided via the femoral artery sheath through the aortic valve and positioned within the left ventricle. Blood samples will then be taken from the left ventricle and CS for cross heart substrate analysis prior to the administration of heparin. The atria will then be paced at a rate just above baseline to ensure a steady rate. The conductance catheter allows continuous and simultaneous sampling of pressure and volume at 4 ms intervals, allowing the acquisition of pressure-volume loops in real-time. CS thermodilution runs will be performed in triplicate (with an average of 3 runs used for analysis). Samples will then be taken for oxygen and carbon dioxide measurement. Oxygen measurement will be performed using a Bayer Rapidlab 800 series blood gas analyser (Bayer Healthcare LLC, East Walpole, MA, USA). Carbon dioxide measurement will be performed by mass spectrometry by the University of Brimingham using a Finnigan MAT Delta^plus^X mass spectrometer. PV loops will be acquired from the left ventricle with a CFL-512 system (CD Leycom), which allows further offline analysis (CircLab, Leiden University, The Netherlands) for determination of left ventricular stroke work (LVSW), mechanical efficiency, dp/dt_max_, dp/dt_min_, and Tau (rate of active relaxation).

The steady heart rate will allow for accurate assessment of per beat myocardial oxygen consumption. All of this subgroup will have had CMR studies for accurate assessment of cardiac mass and thus permit correction for myocardial mass.

Respiratory quotient (RQ) is a unitless value found by dividing the amount of eliminated CO_2 _by the O_2 _consumed [1].(1)

Mechanical efficiency (ME) can be calculated by dividing work output by work input giving another dimensionless value or percentage [2].(2)

Work input can be calculated from myocardial oxygen consumption measurements according to the Fick principle by multiplying coronary sinus blood flow by the ateriovenous oxygen content difference[18]. To be used in the above equation the caloric equivalent of 1 mL of O_2 _is ≈20J will be used[19].

External work will be estimated from measurements taken from the PV loops acquired from the left ventricle. 1 mmHg × mL equates to 1.33 × 10^-4 ^J for the purposes of calculating ME[19].

### Randomisation and Drug Monitoring

Following baseline investigations ensuring normal liver function tests, participants will be randomised by computer to commence with perhexiline 200 mg oral daily or an identical placebo for one month (Figure 1). Randomisation will be done in a 50:50 ratio of perhexiline:placebo in blocks of 10.

At one week duration, all patients will have a blood sample taken for serum perhexiline level with the researcher blinded to the subject's treatment allocation. This level will be evaluated by a doctor unblinded to the subject's treatment allocation. The dosage of perhexiline will be adjusted according to the accepted dosing protocol (Table 2). An equal number of dummy dose adjustments will be made to those patients on placebo to help to maintain blinding.

**Table 2:** Perhexiline dose titration according to serum perhexiline level.

*Perhexiline concentration (mg/L)*	*Recommended new daily dose (mg)*
0.00-0.05	300
0.05-0.15	250
0.15-1.00	200
1.00-1.50	100
1.50-2.00	50
>2.00	Cease for 1 week and start 50 mg alternate days

At the end of one month of therapy, patients will undergo a repeat of baseline investigations and a further perhexiline level will be taken to ensure therapeutic levels were achieved and compliance maintained.

### Endpoints

The primary end point of the study will be an increase in the PCr/ATP ratio as assessed by MRS after 4 weeks of treatment. This end point has been used in previous studies and is associated with symptomatic class and mortality in heart failure[20]. Secondary endpoints are: i) respiratory quotient; ii) mechanical efficiency; iii) change in LV function.

### Planned Statistical Analysis

Comparisons will be performed between the groups at baseline and after the 4 week treatment. The normality of distribution of all continuous variables will be determined using a normality plot and the Kolmogorov-Smirnov test. Normally distributed variables will be analysed using unpaired t-tests, χ^2 ^or repeated measures analysis or variance. Variables not normally distributed will be log transformed prior to analysis to achieve normal distribution or, if this is not achieved, analysed by Mann-Whitney U or Kruskal-Wallis tests.

Analysis will be by intention-to-treat. A *p*-value of < 0.05 will be considered statistically significant.

Sample size calculations were based on the primary end point of change in PCr/ATP ratio after 4 weeks of treatment. Pilot work from our group using the same MRS technique and perhexiline revealed a 0.4 improvement in PCr/ATP ratio in the treatment group by comparing means. The response was normally distributed with a standard deviation of 0.5. Using this data, 26 subjects in each arm will provide at least 80% power of detecting a 0.4 change in PCr/ATP ratio using a two-tailed t-test at the 5% significance level. Recruiting 50 patients will allow for withdrawal, drop out or incomplete data.

### Monitoring and Safety Assessments

When perhexiline levels are unmonitored, it has been associated with both hepatotoxicity and peripheral neuropathy. All patients will be screened both clinically and biochemically for any sign of these conditions. Perhexiline also lowers serum glucose levels. Patients who are diabetic will be made aware that the medication may cause hypoglycaemic and they will be instructed make frequent blood sugar measurements when starting the study. Perhexiline levels will be tested at one week and at the end of the study as outlined above.

All adverse events, including serious adverse events (SAEs), will be recorded and followed up for the duration of the study or until resolution. Assessment of adverse events will be performed by study investigators. All SAEs will be graded and reported to the sponsor. Any suspected unexpected serious adverse reactions will be reported to the sponsor, ethics committee and the Medicines and Healthcare Products Regulatory Agency. This study has been reviewed and approved by the South Birmingham Research Ethics Committee. Written informed consent will be obtained from all study participants.

### Study Limitations

The study is powered for the primary endpoint (an increase in PCr/ATP ratio assessed after 4 weeks of treatment). It is accepted that the study is likely to be underpowered for the secondary endpoint of improvement in LV function. Nonetheless, a similar sized study using perhexiline in heart failure showed a dramatic, absolute increase in ejection fraction of 10% after 2 months of treatment[21]. This improvement had a standard deviation of approximately 10%. Assuming a similar distribution in this study, our sample size should allow us to detect an 8% improvement in ejection fraction, which is comparable to that demonstrated in a recent meta-analysis highlighting the effect of trimetazidine (another metabolic modulating agent) on ejection fraction in non-ischaemic heart failure[22].

No similar invasive work of this nature has been performed in human heart failure to allow an accurate power calculation for this component of the trial.

## Discussion

The finding that perhexiline improves cardiac energetics by an increase in the PCr/ATP ratio would be an important contribution to support the future work of metabolic manipulation as a treatment for heart failure, even in the absence of coronary artery disease. PCr/ATP ratio has been shown to correlate with mortality, and an improvement in this index may also lead to an improved survival in this patient population.

Until now, the proposed mechanism of perhexiline is based on in vitro and animal work. By showing an increase in R/Q we can support the hypothesis of increased carbohydrate metabolism as a result of perhexiline therapy.

Improvement in mechanical efficiency would reveal that the above changes contribute, at least in part, to a direct improvement in cardiac function.

## List of Abbreviations

2,3-DPG: 2,3-diphosphoglycerate; AMARES: advanced method for accurate, robust and efficient spectral fitting; ATP: adenosine triphosphate; CPT: carnitine palmitoyltransferase; BNP: brain natriuretic peptide; CMR: cardiac magnetic resonance imaging; CS: coronary sinus; ECG: electrocardiogram; EF: ejection fraction; FAO: fatty acid oxidation; FWHM: frequency width at half maximum; HF: heart failure; ISIS: image selected in-vivo spectroscopy; jMRUI: java based magnetic resonance user interface; LV: left ventricle; LVSW: left ventricular stroke work; ME: mechanical efficiency; MRS: magnetic resonance spectroscopy; MLWHF: Minnesota Living with Heart Failure; NHYA: New York Heart Association; P-31: phosphorus-31; PCr: phosphocreatine; PV: pressure volume; RQ: respiratory quotient; SAE: serious adverse event; TCA: tricarboxylic acid cycle;

## Competing interests

The sponsor for this trial is University Hospital Birmingham NHS Foundation Trust, Birmingham, UK. Prof MP Frenneaux holds a patent for the use of perhexiline in heart failure.

## Authors' contributions

All authors contributed to the design of the study and were involved in drafting of the protocol and this manuscript. RMB will acquire the majority of the data. All authors will be involved in the statistical analysis and data interpretation. All authors have read and approved the final version of the manuscript.

## References

[B1] Stewart S MacIntyre K Hole DJ Capewell S McMurray JJ More 'Malignant' Than Cancer? Five-Year Survival Following a First Admission for Heart Failure Eur J Heart Fail 2001 3 3 315 22 10.1016/S1388-9842(00)00141-0 11378002

[B2] Brown AM Cleland JG Influence of Concomitant Disease on Patterns of Hospitalization in Patients With Heart Failure Discharged From Scottish Hospitals in 1995 Eur Heart J 1998 19 7 1063 9 10.1053/euhj.1997.0859 9717042

[B3] Khand A Gemmel I Clark AL Cleland JG Is the Prognosis of Heart Failure Improving? J Am Coll Cardiol 2000 36 7 2284 6 10.1016/S0735-1097(00)00995-5 11127474

[B4] Bottomley PA Wu KC Gerstenblith G Schulman SP Steinberg A Weiss RG Reduced Myocardial Creatine Kinase Flux in Human Myocardial Infarction: an in Vivo Phosphorus Magnetic Resonance Spectroscopy Study Circulation 2009 119 14 1918 24 10.1161/CIRCULATIONAHA.108.823187 19332463 PMC2743337

[B5] Neubauer S Krahe T Schindler R Horn M Hillenbrand H Entzeroth C Mader H Kromer EP Riegger GA Lackner K 31P Magnetic Resonance Spectroscopy in Dilated Cardiomyopathy and Coronary Artery Disease. Altered Cardiac High-Energy Phosphate Metabolism in Heart Failure Circulation 1992 86 6 1810 8 1451253 10.1161/01.cir.86.6.1810

[B6] Stanley WC Recchia FA Lopaschuk GD Myocardial Substrate Metabolism in the Normal and Failing Heart Physiol Rev 2005 85 3 1093 129 10.1152/physrev.00006.2004 15987803

[B7] Bouche P Bousser MG Peytour MA Cathala HP Perhexiline Maleate and Peripheral Neuropathy 1 Neurology 1979 29 5 739 43 220563 10.1212/wnl.29.5.739

[B8] Roberts RK Cohn D Petroff V Seneviratne B Liver Disease Induced by Perhexiline Maleate 2 Med J Aust 1981 2 10 553 4 7321956 10.5694/j.1326-5377.1982.tb124213.x

[B9] Cole PL Beamer AD McGowan N Cantillon CO Benfell K Kelly RA Hartley LH Smith TW Antman EM Efficacy and Safety of Perhexiline Maleate in Refractory Angina. A Double-Blind Placebo-Controlled Clinical Trial of a Novel Antianginal Agent 1 Circulation 1990 81 4 1260 70 10.1161/01.CIR.81.4.1260 2180591

[B10] Fragasso G Perseghin G De CF Esposito A Palloshi A Lattuada G Scifo P Calori G Del MA Margonato A Effects of Metabolic Modulation by Trimetazidine on Left Ventricular Function and Phosphocreatine/Adenosine Triphosphate Ratio in Patients With Heart Failure Eur Heart J 2006 27 8 942 8 16510466 10.1093/eurheartj/ehi816

[B11] Abozguia K Elliott P McKenna W Phan TT Nallur-Shivu G Ahmed I Maher AR Kaur K Taylor J Henning A Ashrafian H Watkins H Frenneaux M Metabolic Modulator Perhexiline Corrects Energy Deficiency and Improves Exercise Capacity in Symptomatic Hypertrophic Cardiomyopathy Circulation 2010 122 16 1562 9 10.1161/CIRCULATIONAHA.109.934059 20921440

[B12] Vanhamme L Sundin T Hecke PV Huffel SV MR Spectroscopy Quantitation: a Review of Time-Domain Methods NMR Biomed 2001 14 4 233 46 10.1002/nbm.695 11410941

[B13] Conway MA Bottomley PA Ouwerkerk R Radda GK Rajagopalan B Mitral Regurgitation: Impaired Systolic Function, Eccentric Hypertrophy, and Increased Severity Are Linked to Lower Phosphocreatine/ATP Ratios in Humans Circulation 1998 97 17 1716 23 9591766 10.1161/01.cir.97.17.1716

[B14] Schiller NB Shah PM Crawford M DeMaria A Devereux R Feigenbaum H Gutgesell H Reichek N Sahn D Schnittger I Recommendations for Quantitation of the Left Ventricle by Two-Dimensional Echocardiography. American Society of Echocardiography Committee on Standards, Subcommittee on Quantitation of Two-Dimensional Echocardiograms J Am Soc Echocardiogr 1989 2 5 358 67 2698218 10.1016/s0894-7317(89)80014-8

[B15] Lang RM Bierig M Devereux RB Flachskampf FA Foster E Pellikka PA Picard MH Roman MJ Seward J Shanewise JS Solomon SD Spencer KT Sutton MS Stewart WJ Recommendations for Chamber Quantification: a Report From the American Society of Echocardiography's Guidelines and Standards Committee and the Chamber Quantification Writing Group, Developed in Conjunction With the European Association of Echocardiography, a Branch of the European Society of Cardiology 1 J Am Soc Echocardiogr 2005 18 12 1440 63 10.1016/j.echo.2005.10.005 16376782

[B16] Nagueh SF Appleton CP Gillebert TC Marino PN Oh JK Smiseth OA Waggoner AD Flachskampf FA Pellikka PA Evangelista A Recommendations for the Evaluation of Left Ventricular Diastolic Function by Echocardiography J Am Soc Echocardiogr 2009 22 2 107 33 10.1016/j.echo.2008.11.023 19187853

[B17] Alam M Wardell J Andersson E Samad BA Nordlander R Characteristics of Mitral and Tricuspid Annular Velocities Determined by Pulsed Wave Doppler Tissue Imaging in Healthy Subjects J Am Soc Echocardiogr 1999 12 8 618 28 10.1053/je.1999.v12.a99246 10441217

[B18] Fick A Uber Die Messung Den Blutquantums in Der Herzventrikeln Sitzungb Phys Med Gez Wurzberg 1870 16

[B19] Gibbs CL Cardiac Energetics Physiol Rev 1978 58 1 174 254 146205 10.1152/physrev.1978.58.1.174

[B20] Neubauer S Horn M Cramer M Harre K Newell JB Peters W Pabst T Ertl G Hahn D Ingwall JS Kochsiek K Myocardial Phosphocreatine-to-ATP Ratio Is a Predictor of Mortality in Patients With Dilated Cardiomyopathy Circulation 1997 96 7 2190 6 9337189 10.1161/01.cir.96.7.2190

[B21] Lee L Campbell R Scheuermann-Freestone M Taylor R Gunaruwan P Williams L Ashrafian H Horowitz J Fraser AG Clarke K Frenneaux M Metabolic Modulation With Perhexiline in Chronic Heart Failure: a Randomized, Controlled Trial of Short-Term Use of a Novel Treatment Circulation 2005 112 21 3280 8 10.1161/CIRCULATIONAHA.105.551457 16301359

[B22] Gao D Ning N Niu X Hao G Meng Z Trimetazidine: a Meta-Analysis of Randomised Controlled Trials in Heart Failure Heart 2011 97 4 278 86 10.1136/hrt.2010.208751 21134903

